# Management of diabetes mellitus at the household level using community health strategy in Embu County, Kenya

**DOI:** 10.11604/pamj.2021.39.35.27753

**Published:** 2021-05-12

**Authors:** Salim Ali Hussein, Peter Kithuka, George Otieno, Alison Yoos, Rosaline Kaugi, Cecelia Njeru

**Affiliations:** 1 Department of Health Management and Informatics, School of Public Health, Kenyatta University, Nairobi, Kenya,; 2 Improving Public Health Management for Action (IMPACT), Nairobi, Kenya,; 3 National Government, Ministry of Health, Nairobi, Kenya,; 4 Department of Health, Embu County Government, Embu, Kenya

**Keywords:** Diabetes management, health system, knowledge, community health strategy, community health volunteers, community health units, non-communicable diseases

## Abstract

**Introduction:**

diabetes mellitus (DM) causes 1% of the mortality in Kenya and 2% of the population in Kenya has diabetes mellitus. Embu County was the 5^th^ leading county in diabetes mellitus morbidity in Kenya in 2019. This study aimed at assessing the management of diabetes mellitus at the household level using the community health strategy in Embu County. Community health strategies in the county is implemented using Community Health Volunteers.

**Methods:**

it was a cross-sectional study using 422 household participants in Embu County and 150 community health volunteers´ (CHVs). Key informant interviews were used on community health strategy managers involved in diabetes management programmes in the County. Data was analyzed using SPSS version 25.

**Results:**

factors that were positively associated with effective management of DM at household level in the County were financial support and supervision of community health units, provision of tools and commodities, use of health information system, training of level of CHVs in management of hypertension and diabetes mellitus and subsequent knowledge on symptoms, defining and classifying DM, treatment, prescription of drugs, knowledge of risk factors of DM and prevention of DM at household level.

**Conclusion:**

success of utilization of community health strategies for diabetes management will require adequate training of CHVs in management of hypertension and diabetes mellitus, financial support of community health units, adequate supervision of community health units, financial support of community health units, provision of tools and commodities and community health units (CHU) utilizing health information system.

## Introduction

Diabetes mellitus (DM), also referred to as diabetes, is a chronic condition that results due to elevated levels of glucose in blood caused by the body´s inability to produce any or enough of the hormone insulin or use insulin effectively [1]. This lack of insulin production or the failure of body cells to utilize it resulting in hyperglycaemia which is the hallmark of diabetes [2]. Diabetes is categorized into three main types: type 1, type 2 and gestational diabetes (GDM). Diabetes type 1 arise from an autoimmune reaction caused by the body´s immune cells destroying the pancreases beta cells responsible for insulin production resulting in a relative or absolute deficiency of insulin as the pancreases produces very little to none of the hormone. On the other hand, elevated blood glucose occurring in diabetes type 2 also called insulin resistance, is initiated by the body´s inability to fully respond to insulin or the body producing insufficient volumes of this hormone. Conversely gestational diabetes mellitus (GDM) is hyperglycaemia that is first detected during pregnancy [2]. In 2015, 75% of all NCD related death occurred in low-and middle-income countries (LMIC) [3], where the prevalence of diabetes had quadrupled between 1980 and 2014 [2]. This skyrocketing rise in the prevalence of NCDs which has also affected the most economically active age group, has had negative consequences on health systems and populations since a large percentage of the health care system in the world is poorly prepared to deal with prevention and management of NCDs [4]. Studies involving non-communicable diseases (NCDs) such as diabetes and hypertension have shown community health volunteers (CHVs) play a crucial role in the prevention and management of this chronic condition [5]. Similarly, their role in the prevention and management of diabetes and hypertension in resource limited countries has been noted [6]. CHVs have been found to be effective in providing health education, encouraging adherence support and counselling leading to better outcomes in terms of early detection, prevention and management of NCDs [7]. This is because they act as important link and intervention between the community and the healthcare system as they aid in provision of a context-specific support leading to better long-term outcomes for the participants and community.

Callaghan M *et al*. [8], found that the performance of community health volunteers is dependent on the interface between their motivation and constraints that they face in their work. The motivations can be intrinsic including monetary incentives, work environment, and remuneration as well as extrinsic motivators which include training, supportive supervision, appreciation by managers, colleagues, and the community. A study conducted by Jeet G *et al*. [9] established that CHVs operate effectively in environment full of support, mutual respect and trust from other cadre of health workers. Findings from Aponte J *et al*. [10] found that performance of community health workers is affected by experience in practice when combined with their positive attitude towards their undertaking. Implementation of community health strategy require adequate supervision, adequate commodities, materials and equipment [4, 11]. Muriuki D *et al*. [12] found that for CHVs to be effective in management of NCDs they need to have adequate knowledge of risk factors, complication, prevention and treatment. The utilization of community health volunteers (CHVs) has been identified as one of core strategy to address the challenge of shortage of health workers in developing nations [3]. In Kenya, community health strategy has been adopted in the 47 counties as a component of cost cutting measures in addressing health needs of communities and increasing accessing to health services. Embu County has been preparing for Universal Health Coverage (UHC) by implementing community health strategies through utilization of community health workers (CHVs) [11].

The county has achieved the envisaged 10 CHVs per Community Health Unit (CHU), serving a population of about 5,000 (1,000 households). While financing of community health strategies remains a key challenge in most of the counties in Kenya, Embu County has taken positive steps towards financing Community Health Services (CHS) with a budget of 50 million for period of between 2018-2022) [13]. CHVs in Embu County are regularly trained using Kenya health model for quality health on how to manage regular pandemics in the County [13]. Despite the enormous budgetary support, training and experience with CHV, Embu County continues to experience Non communicable diseases burden of unprecedented level touching on the role of primary health care in the county. The prevalence and burden of Non-communicable Diseases (NCDs) such as Diabetes and Hypertension is 5.6 per cent in Embu County. The rise in prevalence has been a major concern on effectiveness of community health strategy in management of diabetes mellitus in various households in the County [13]. The common questions have been why they has been increasing in the burden of NCDs, despite the county´s heavy investment in community health strategies through use of CHVs. The focus of the study is to examine factors in which CHVs´ interventions operate in diabetes management at the household level in the Embu County. Information gained from this study will aid in coming up with strategies of improving community health Volunteers´ performance and coming up with policies on better management of diabetes at household level.

## Methods

### Study setting

This study was conducted in Manyatta constituency of Embu County. Embu is one of the 47 counties of Kenya, it has four constituencies, Manyatta, Runyenjes, Mbeere South and Mbeere North. Of the four constituencies, Manyatta constituency is the most densely populated with a projected population of 177,159 and population density of 659 per square kilometers. The constituency is made of seven locations: Gaturi south, Kithimu, Mbeti North, and Embu municipality, Ngandori, Nginda and Ruguru. The county has achieved the envisaged 10 CHVs per Community Health Unit (CHU), serving a population of about 5,000 (1,000 households). CHVs serve at the village level with approximate of 100 households. They are required to provide regular medical reports and feedback to a nearby community health unit usually at the sub-county level. Embu County has an increase in the burden of NCDs, despite the county´s heavy investment in community health strategies [13].

### Study design

This study adopted a cross-sectional survey study design. It then used quantitative and qualitative approaches in assessing management of diabetes mellitus at the household level using the community health strategy. DM in Manyatta constituency of Embu County. The study included resident of the study area who were aged above 18 years have lived in the areas for at least one year and who consented to take part in the study. For the key informants, the study included all CHVs who were actively involved in diabetes programs and were managing diabetes in their communities, who consented to take part in the study. We excluded those who had not actively participated in the management of diabetes in in the preceding 1 year.

### Sample size and sampling procedure

To select the households to be used in the study, first, the constituency was divided based on the number of locations, Manyatta constituency has 7 locations. The number of households to be sampled in each location was then equally distributed among the locations (422/7 = 60). The 60 households sampled in each location was equally distributed among villages in each location. Using a sampling frame of houses in each village, convenience sampling was used to select the households that participated in the study. This sampling was done to represent all socio-economic classes. A head of household who met the study inclusion criterion and agreed to participate in the study was interviewed. Purposive expert sampling was used in selecting key informants. This method has been found to best suited when the aim of the research is to obtain information from individuals that have better understanding, or particular expertise of the issue being studied [14]. This were mainly the health care facility administrators and medical officers working in government health facilities operating within the study area, in total 12 KIIs were interviewed. Purposive sampling was used to select all the 30 CHVs that had been trained on DM management at the household level and were interviewed. These CHVs were operating in each of the seven locations chosen for the study.

### Study tools and data collection

A diabetes management knowledge assessment questionnaire was administered orally by trained enumerators for both households and CHVS. The questionnaires contained questions assessing signs, symptoms, and prescription of diabetes mellitus. It was adopted from a study on national strategy for the prevention and control of non-communicable disease and the questions were modified using the CHVS training manuals provided by the Embu County strategy for the prevention and control of non-communicable disease [14]. A trained data collector was used to score on how CHVs manages diabetes in the study area, they were also required to observe and score the process of management of diabetes at household. The CHVs were expected to demonstrate an ability to identify the signs and symptoms of these illnesses as well as prescribe proper medications.

### Measurement of diabetes management

As an outcome variable, management of DM at household level was defined as taking caring for patients with diabetes mellitus at home to eliminate symptoms and to prevent, or at least slow, the development of complications, through behavior change, appropriate referral, and community diagnosis of DM, eating balanced diet, lowering salt and sugar, eating fruits and vegetables, DM education and adherence to DM medication. In the study, paper assessed community health strategy on the management of DM at the household level by combining scores from diabetes management outcomes which includes adopting lifestyle changes including physical exercises, identifying symptoms and how to prevent it, behavior change on smoking and alcohol intake, stress management, knowing of risk factors of diabetes referral, community diagnosis of DM and adherence to DM medication which were converted to percentages and then categorized into good, moderate, and poor scores. Aggregate score was arrived at by total score based on a scale of 6 parameters measuring Diabetes management on a two scale where; 1- Yes, 2-No, which were multiplied by 50 questions scale, therefore the highest or maximum possible score or level for Diabetes management was 100 score (50x2). A score of 0-49% was classified as poor, 50-74% as moderate, and 75-100 score as good. The combined scores from the diabetes management and case scenarios were aggregated into two categories: below 50% and those above 50%.

### Data collection and analysis

Statistical Package for the Social Sciences (SPSS) version 25 was used in data analysis. Descriptive statistics (frequencies, bar graphs and percentages) were used to describe, organize, and summarize collected data. Logistic regression was used to determine community health strategies associated with diabetes management at household level. Chi-square from Cross tabulations was used to assess the relationship between two variables. A p value of < 0.05 will be set as statistical significance. The multivariable analysis shows AORs with corresponding 95% CIs.

### Ethics approval

Approval to conduct the study was obtained from the following bodies: Board of Postgraduate Studies of Kenyatta University, Kenyatta university ethical review committee, NACOSTI and the administration of Embu County. Signed informed consent was required from each participant prior to enrolment in the study. Participant identifiers was used in the study instrument to protect the identity of study participants. No one was enrolled or interviewed prior to giving signed informed consent.

## Results

A total of 452 respondents participated in the study including 422 households´ members and 30 CHVs. Seventy-two percent (305) of them were female and 28.0% (117) were male. Their mean age was 40 years, and their primary occupation was farming (62%) trading and casual laborers. In all, 78% of them were married and 58% had graduated from post primary school (Table 1).

**Table 1:** demographic characteristics of 452 respondents who participated in the study including 30 CHVs and 422 household members

	Frequency	Percentage
**Gender**		
Male	117	27.7
Female	305	71.8
**Age**		
18-30 Year	82	19.4
31-40 Year	114	27.0
41-50 Years	103	24.4
Over 50	123	29.1
**Education**		
Lack formal Education	26	6.2
Primary	185	43.8
Post primary education	211	50.0
**Occupation**		
Formal Employment (Salaried)	8	2.1
Informal Employment (Casual)	414	97.9
**Distance to health facility**		
Less than 1 KM	203	48.1
1-5 KM	147	34.8
Above 5 KM	70	17.1
**Means of transport**		
Foot	288	71.5
Bicycle or motorbike	46	10.9
Motorized	43	10.2
Mixed	59	14.6
Non-Response	19	4.5
**Income Group**		
Less than 10,000	268	63.5
10,000-20,000	64	17.2
Above 20,000	26	5.8
Non-Response	64	15.2

### Management of diabetes at household level

The study found that overall, the management of diabetes at household level was low. There were 252 household (60.7%) with poor scores in management of diabetes, while 70 (16.5%) had moderate scores, and 100 (23.6%) had good scores. Majority of household members had poor score in terms of adopting lifestyle changes including physical exercises, eating balanced diet, lowering salt and sugar, eating fruits and vegetables, identifying symptoms and how to prevent it, knowing of risk factors of diabetes referral, community diagnosis of DM and adherence to DM medication. In terms of knowledge of hypertension and diabetes mellitus for effective management at households, 10 respondents (33.0%) had poor scores, 15(50.0%) had moderate scores, and only 5(16.6%) had above average scores. Lowest overall diabetes management scores at households were found to be in behavior change in terms of cessation or moderation of smoking and alcohol intake, stress management and adherence to DM medication (Table 2).

**Table 2:** diabetes management scores of sampled 422 household´s members in Embu County

	Frequency	Percent
Low Score of Diabetes Management	253	60.7
High Score of Diabetes Management	169	29.33
Scores for Diabetes Management at Households		
Identifying signs and symptoms of diabetes	34	
Risk factors of diabetes	46	
Eating Healthy	25	
Limiting and stopping smoking	57	
Control of what they drink	25	
Avoiding stress	34	
Regular physical exercise	47	

### Factors associated with management of diabetes mellitus at the household level

For better delivery and outcomes of diabetes management it requires community health strategies that are effective. Community health strategies in the county is implemented using CHVs. Community Health Volunteers (CHVs) are an important link between the community and healthcare, an intervention program or service delivery through provision of a context-specific support leading to better long-term outcomes for community health strategies This study aimed to assess the management of diabetes mellitus at the household level using the community health strategy in Embu County. Bivariate analysis was performed to determine the odds ratios (CORs) of obtaining good outcomes of diabetes management after implementation of community health strategies in the County. The CHVs who were supervised were more likely to score above 50% in terms of DB Management outcomes in the households as compared to those who were not supervised (AOR 5.00; 95% CI 4.80-5.23). CHU that was funded adequately produced higher scores of diabetes management of above 50% (AOR 2.23; 95% CI 2.00-2.30) than those who had lower or no funding in a period of time. CHU that was provided with adequate tools and commodities produced higher scores of diabetes management of above 50% (AOR 4.00; 95% CI 3.80-4.12) than those who had lower or no funding in a period of time. The CHUs which were utilizing community-based health information systems (CBHIS), were more likely to score above 50% (AOR 3.9; 95% CI 3.80-4.54) than those who were not utilizing the system.

The CHVs whose had received training on how to counsel households´ members on self-management of diabetes mellitus were more likely to produce better outcomes of diabetes management than those who had not received training (AOR 2.1; 95% CI 1.54-2.41). The CHVs who served 150-300 households were less likely to score above 50% (AOR 4.24 95% CI 4.12-5.50) compared with those who served 150 or less. Similarly, CHVs serving more than 200 households were less likely to have scores above 50% compared with those who were serving 100 or less households (COR 0.24; 95% CI 0.13-0.46). After the assessment it was found that CHVs who scored above 50.0% in terms of knowledge on defining and classifying DM, treatment, prescription of drugs, knowledge of risk factors of DM and prevention of DM were more likely of producing better outcomes of diabetes management at various households as compared to those who were had lower scores below 50.0% (AOR 2.73; 95% CI 1.50-4.92) (Table 3).

**Table 3:** chi-square results of community health strategies adopted in Embu County versus diabetes management scores of samples 422 household´s members in Embu County

				Odd Ratio	95% Confidence Interval for Exp(B)	
					Lower Bound	Upper Bound
	Intercept	Chi-Square	Sig			
**CHVs knowledge of defining and classifying DM**	Yes	14.4	0.001	13.9	13.5	14.2
	No			11.2	11.0	11.5
**CHVs knowledge of signs and symptoms**	Yes	11.2	0.020	20.0	19.7	20.8
	No			8.90	8.90	9.20
**CHVs knowledge of risk factors of DM**	History of diabetes	11.1	0.110	8.90	8.88	9.20
	Pregnant women			11.20	11.00	11.30
	Age			8.90	8.80	9.10
	Blood pressure			2.00	1.97	2.10
**CHVs knowledge of prevention of DM**	Yes	11.2	0.420	8.90	8.88	9.20
	No			1.00	0.89	1.30
**CHVs knowledge of treatment of DM**	Life>	3.12	0.560	11.0	9.87	11.5
	Oral medication			8.2	8.00	8.5
	Insulin injections			4.2	3.8	4.5
	Comorbidity			12.0	11.8	12.4
**CHVs knowledge of identifying complications**	Eye/vision problem		0.00	11.0	10.73	11.5
	Heart disease	14.3		3.20	3.20	3.50
	Stroke			2.51	1.482	2.81
	loss of limbs			5.00	4.70	5.40
**Level of supervision of community health units**	Yes	13.49	0.000	5.0	4.80	5.23
	No			1.2	.080	1.30
**Financial support of community health units**	Yes	11.90	020	2.23	2.00	2.30
	No			1.00	081	1.30
**Health kits and provision of tools and commodities**	Yes	12.00	020	4.00	3.80	4.12
	No			1.11	0.91	1.34
**CHU use of health information system**	Yes	15.90	020	3.9.	3.80	4.54
	No			1.5	1.1	2.00

Significance level at α=0.05

## Discussion

Diabetes management in the study area is inadequate. The manner in which community health strategies is organized and delivered at community health unit encompasses supervision of community health unit, health system financing, CHUs commodities and CHVs kits, availability and utilization of health information system was seen to have direct influence on level of diabetes management in the study area. The level of supervision at CHU level was seen as ways of improving the management of diabetes at household level. Studies have shown that support supervision is critical for improving the performance of CHVs (11). However, despite its importance, the implementation of support supervision has been identified as a weak link in many CHV programs [6].

The study found that level of motivation of CHU and CHVs was associated with diabetes management outcomes, this was in terms of either financial incentives as well as provision of tools and commodities. This agrees with study by [11] found that for effective service of CHVs, the relationship between them and professional health workers should be characterized by support, mutual respect, trust and partnerships. The health professions in the study respected and supported CHV since they collaborate with them and they are the ones who are playing important roles in areas where there is limited access to professional health care [1]. The study found that level of training of CHVs was associated with DB management outcomes such that for CHVs to be effective in executing their task, they must have adequate training, supportive supervision, materials and equipment. The CHVs whose had received training on how to counsel households´ members on self-management of diabetes mellitus were more likely to produce better outcomes of diabetes management to those who had not received training. Training community health workers has been identified as critical element of improving their operations, whereby the more intensive the training the better the outcomes [9].

The CHVs who were utilizing community-based health information systems (CBHIS), were more likely to score above 50% (AOR 3.9; 95% CI 3.80-4.54) than those who were not utilizing the system. After the assessment it was found that CHVs who scored above 50.0% in terms of assessment of level of knowledge on defining and classifying DM, treatment, prescription of drugs, knowledge of risk factors of DM and prevention of DM produced better outcomes of DB Management at various households as compared to those who were had lower scores below 50.0% (AOR 2.73; 95% Ci 1.50-4.92). A study by Dube L *et al*. [6] found that effectiveness of community health workers requires knowledge of risk factors, complication, prevention and treatment to be effective in executing their task.

## Conclusion

Diabetes management in the study area is inadequate, support supervision and the recruiting of CHVs with at least basic -level education may be helpful in improving the performance of CHVs. For better delivery and outcomes of diabetes management there should be programmes that incorporate CHVs. Quality supervision of entire community health unit is critical component of community health strategy. For effective community health strategy to succeed there is a need for adequate financial support, provision of adequate commodities, adequate training of CHVs, supportive supervision, materials and equipment. It is paramount to recognize the key characteristics of CHVs as well as their required training and level of support that are required so as to enable the CHVs work effectively in their communities. Supervisors of community health units need to ensure that the CHVs´ workload is moderated, and one key strategy is to ensure that they serve less than 100 households. Short training programs are beneficial but not sufficient and should be followed up with regular refresher training.

### What is known about this topic

- Kenya struggles with high burden of NCD which diabetes mellitus which is the focus of the study where lack of diagnosis of a high percentage of diabetes cases results in irreversible complications that impose huge financial burdens on the affected individuals CHUs commodities and CHVs kit;
- Kenya has a dire shortage of resources need to address the added diabetes burden, to compound on this, areas where health care workers exist, they are inadequately trained or equipped to appropriately manage diabetes and its related complications;
- There is lack of capacity for early detection of diabetes exists among many health facilities in the country which include routine screening of high blood sugar.

### What this study adds

- The study findings reveal that the success of community health strategy requires combination of health system financing, adequate training of CHVs in management of hypertension and diabetes mellitus, adequate supervision of community health units, financial support of community health units, provision of tools and commodities and CHU utilizing health information system;
- The study findings reveal that role of supervision of CHU as a key strategy in management of Diabetes, since it determines the level program ownership, mentorship, and implementation of health guidelines;
- The study revealed that adequate knowledge of management of hypertension and diabetes mellitus of diabetes is needed both by CHVs as well as household members if the outcome of diabetes management are to be effective.
